# Supplementary figures and images for: Direct and Inverted Repeat stimulated excision (DIRex): Simple, single-step, and scar-free mutagenesis of bacterial genes
Source: PLoS One. 2017 Aug 30;12(8):e0184126. doi: 10.1371/journal.pone.0184126 (PMC5576700; doi:10.1371/journal.pone.0184126)

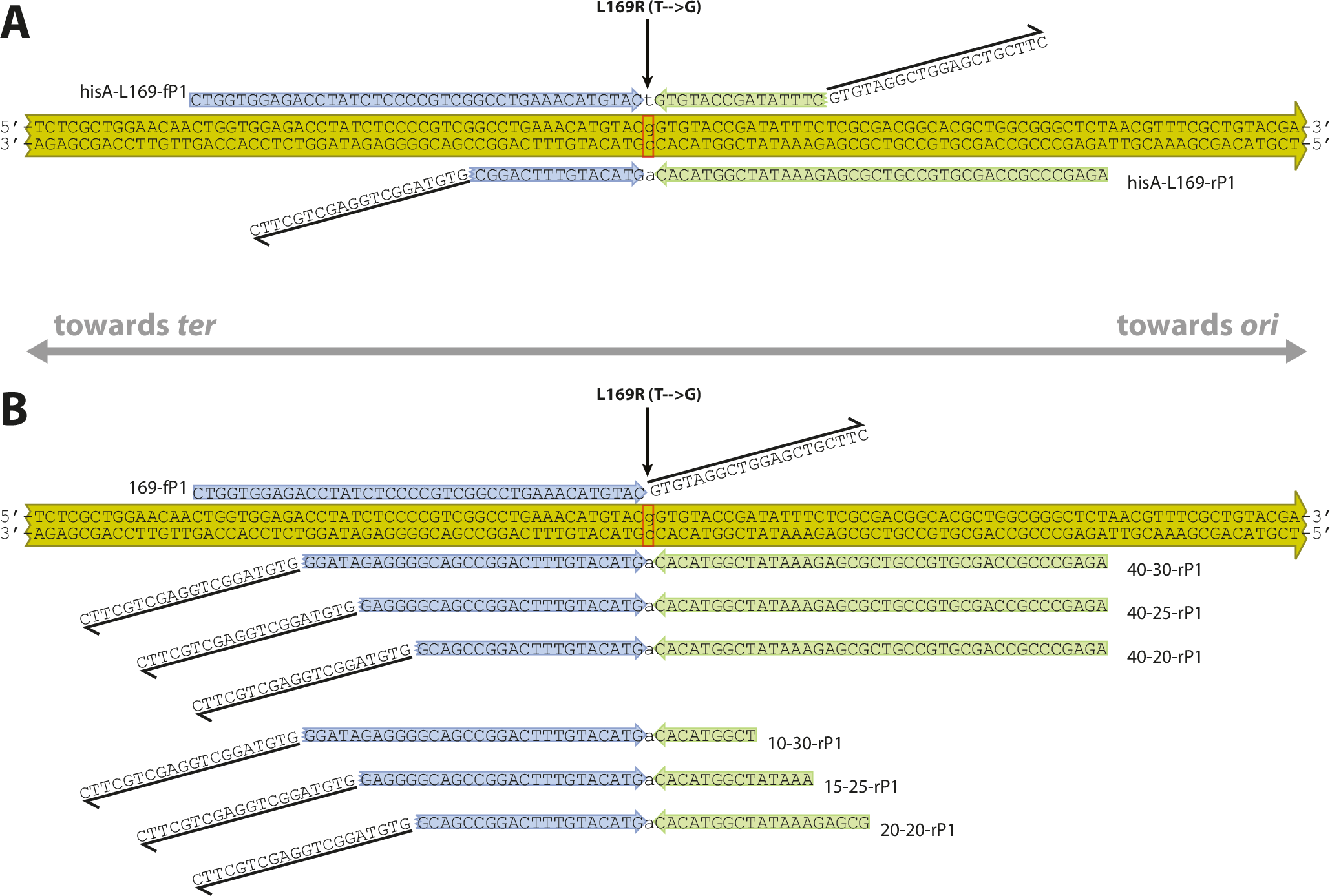

Supplement: S1 Fig — Oligos are aligned to their homologous sequences in the Salmonella enterica hisA gene. Part of the coding sequence of hisA is shown as a double-stranded sequence on top of a yellow arrow, showing the direction of the gene. The vertical arrow points towards the position of the L169R (CAG to CGG) mutation, which is also highlighted with a red box and lower case letters. The green and blue arrows correspond to the “modules” of the recombinogenic ends that are highlighted in green and blue in Fig 6. The slanted”half-arrow” sequences indicate the template annealing portions, which are identical in all oligos. (A) Primers used for the constructs used for the experiments described in Figs 4 and 6B. The overlap between the “upper” primer (hisA-L169-fP1) and the “lower” primer (hisA-L169-rP1) generates the DR in the transformants. (B) Alternative primers used for the constructions in Fig 6D and 6E. The upper primer (169-fP1) was used with one of the lower primers (NN-MM-rP1) to generate different sized DRs from the sequences that overlap between the lower and upper primer, and different amounts of homology to the “left” of the mutation. The design of the oligos used for the constructs in Fig 6C is similar to 40-25-rP1 and 169-fP1, but target the opposite strands to place the cassette on the other side of the mutation. (TIF) [file pone.0184126.s001.tif]

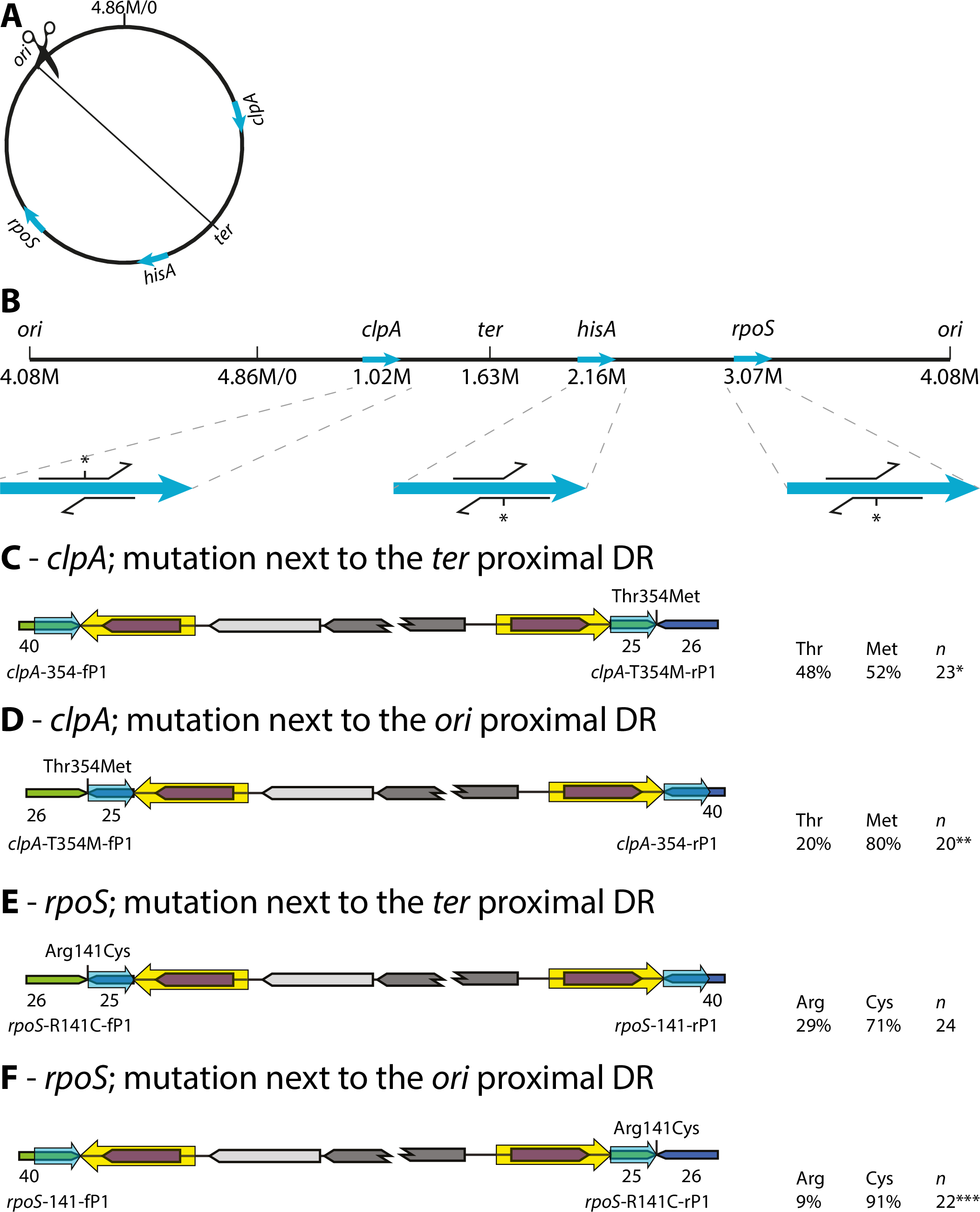

Supplement: S2 Fig — (A) Circular map of the S. enterica chromosome with the directions and approximate locations of the ori and ter and the three genes clpA, hisA and rpoS. (B) Linear representation of the S. enterica chromosome, starting and ending at ori. Zoomed in regions show the individual clpA, hisA and rpoS genes as light blue arrows. The most efficient locus specific primers are indicated, with the mutation-containing primer indicated with an asterisk (these oligos were used in panel D and F in S2 Fig and Fig 6D and 6E. Note that in all three examples the mutation is on the oligo whose homology region is directed towards the ori proximal side. (C) Transformation to construct a Thr354Met mutation in clpA, using a DIRex intermediate with the mutation in the ter proximal DR. (*) Out of 24 sequenced SucS, white segregant clones, one had a ~400 bp deletion in clpA and was discarded. (D) Transformation to construct the same mutation as in (C), but using a DIRex intermediate with the mutation in the ori proximal DR. (**) Out of 24 sequenced SucS, white segregant clones, one had a frameshift mutation in a neighboring codon and was discarded. Three produced poor sequence but was not further tested. (E) Transformation to construct an Arg141Cys mutation in rpoS, using a DIRex intermediate with the mutation in the ter proximal DR. (F) Transformation to construct the same mutation as in (E), but using a DIRex intermediate with the mutation in the ori proximal DR. (***) Out of 24 sequenced SucS, white segregant clones, two had frameshift mutations in neighboring codons and were discarded. The direction of transcription of both genes is left to right in the picture. (TIF) [file pone.0184126.s002.tif]

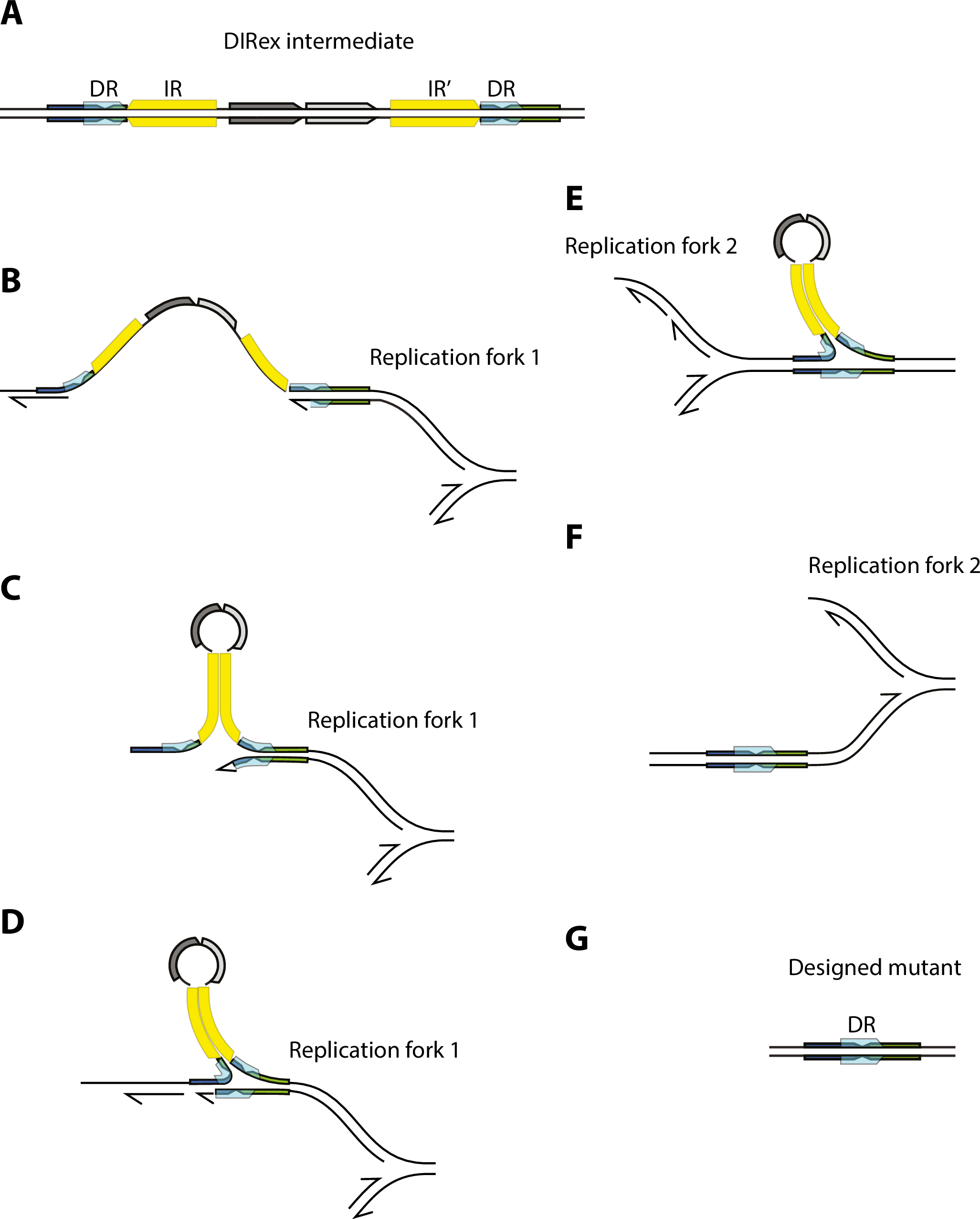

Supplement: S3 Fig — (A) A DIRex intermediate. DRs are indicated as turquoise boxes and IRs as yellow boxes. IR’ indicates the reverse complement of IR. (B) Complementary sequences from IR and IR’ may be exposed during passage of a replication fork, depicted here as a long continuous gap between Okazaki fragments on the lagging strand. (C) Transient intra-strand basepairing between IR and IR’ forms a large stem-loop, stalling replication and bringing a DR and its complementary sequence next to each other. (D) Pairing of the DR and its complementary sequence allows continued lagging strand synthesis. (E) After passage of the first replication fork the new leading strand template lacks the DIRex intermediate and has only one copy of the DR sequence. (F) After passage of the next replication fork, the replicated leading strand carries the designed mutation. (G) The designed mutation segregates into one of the daughter cells during cell division. The model is essentially as suggested by Bzymek and Lovett [31] for stimulation of deletion by uninterrupted palindromes through misalignment during lagging strand synthesis. (TIF) [file pone.0184126.s003.tif]

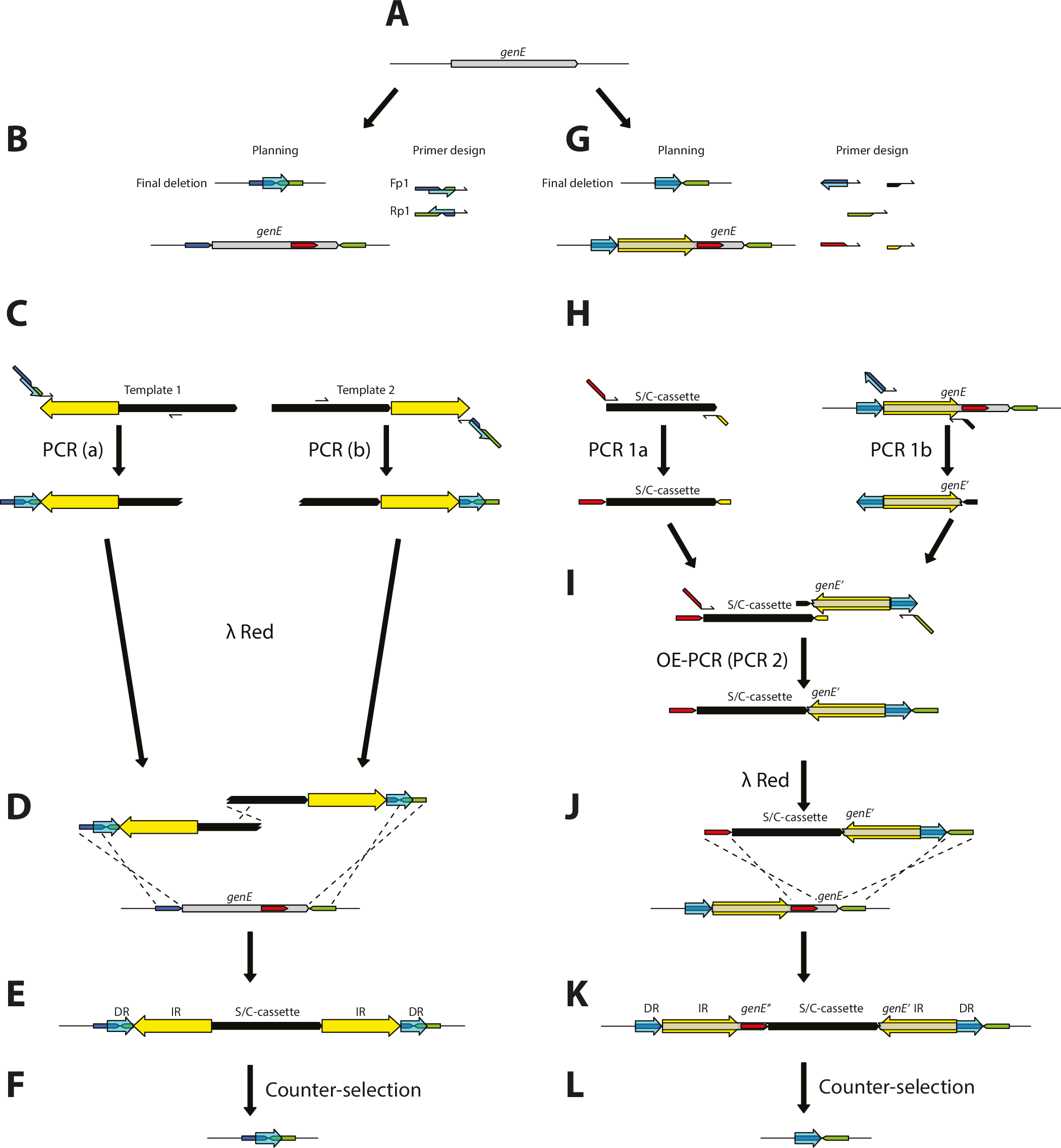

Supplement: S4 Fig — Both methods are used for the construction of the same precise deletion of the hypothetical gene “genE”. (B–F) Use of DIRex for deletion of a gene. (B) Only two gene-specific oligos are needed, in combination with two cassette-specific primers. Recombinogenic tails of the locus-specific primers are chosen so that both oligos contain the designed deletion junction, in this case bringing the “blue box” sequence next to the “green box” sequence and deleting the entire “genE” sequence. The DRs will thus consist of part of the “blue box” and part of the “green box”. (C) Two separate PCR reactions are used to amplify two overlapping “half-cassettes”, each containing one copy of the ~800 bp IR sequence, as well as complementing portions of a selectable and counter selectable (S/C) cassette. (D) The two PCR products are mixed in equimolar amounts and transformed into λ Red induced cells. (E) A semi-stable DIRex intermediate is formed. The S/C cassette (black) is flanked by two inverted repeat sequences (yellow, IR), which in turn are flanked by directly repeated sequences (turquoise, DR), each containing the deletion junction. (F) The final deletion mutant is isolated through selection against the S/C cassette. (G–L) The method described by Tear et al. [19] for deleting a gene. (G) Five specifically designed primers are needed. In addition to generating locus-specific DRs, locus-specific IRs has to be chosen, which limits the method into only constructing deletions and limits the minimal deletion size to the size of the IRs. (H) Two PCR reactions generates the S/C cassette and an artificial locus derived IR-DR cassette. (I) The overlapping S/C-cassette and IR-DR cassette is joined through overlap extension PCR (OE-PCR). (J) The OE-PCR product is transformed into λ Red induced cells. (K) A semi-stable intermediate, analogous to the DIRex intermediate in (E) is formed. (L) The final deletion mutant is isolated through selection against the S/C cassette. Note that the end resu [file pone.0184126.s004.tif]
